# Ultrasound Prevalence and Clinical Features of Nonalcoholic Fatty Liver Disease in Patients with Inflammatory Bowel Diseases: A Real-Life Cross-Sectional Study

**DOI:** 10.3390/medicina59111935

**Published:** 2023-11-01

**Authors:** Ludovico Abenavoli, Rocco Spagnuolo, Giuseppe Guido Maria Scarlata, Emidio Scarpellini, Luigi Boccuto, Francesco Luzza

**Affiliations:** 1 Department of Health Sciences, University “Magna Graecia”, Viale Europa, 88100 Catanzaro, Italy; spagnuolo@unicz.it (R.S.); giuseppeguidomaria.scarlata@unicz.it (G.G.M.S.); luzza@unicz.it (F.L.); 2 Translationeel Onderzoek van Gastroenterologische Aandoeningen (T.A.R.G.I.D.), Gasthuisberg University Hospital, KU Leuven, Herestraat 49, 3000 Leuven, Belgium; emidio.scarpellini@med.kuleuven.be; 3 Healthcare Genetics and Genomics Doctoral Program, School of Nursing, College of Behavioral, Social and Health Sciences, Clemson University, Clemson, SC 29631, USA; lboccut@clemson.edu

**Keywords:** Crohn’s disease, ulcerative colitis, liver steatosis, hepatic ultrasound, metabolism

## Abstract

*Background and Objectives*: Inflammatory bowel disease (IBD) is a condition characterized by chronic intestinal inflammation. We can identify two major forms: Crohn’s disease (CD) and ulcerative colitis (UC). One of the extraintestinal manifestations of IBD is nonalcoholic fatty liver disease (NAFLD). IBD and NAFLD share common pathogenetic mechanisms. Ultrasound (US) examination is the most commonly used imaging method for the diagnosis of NAFLD. This cross-sectional observational retrospective study aimed to evaluate the US prevalence of NAFLD in IBD patients and their clinical features. *Materials and Methods*: A total of 143 patients with IBD underwent hepatic US and were divided into two different groups according to the presence or absence of NAFLD. Subsequently, new exclusion criteria for dysmetabolic comorbidities (defined as plus) were applied. *Results*: The US prevalence of NAFLD was 23% (21% in CD and 24% in UC, respectively). Most IBD–NAFLD patients were male and older and showed significantly higher values for body mass index, waist circumference, disease duration, and age at onset than those without NAFLD. IBD–NAFLD patients showed a significantly higher percentage of stenosing phenotype and left-side colitis. Regarding metabolic features, IBD–NAFLD patients showed a significantly higher percentage of hypertension and IBD plus dysmetabolic criteria. Also, higher values of alanine aminotransferase and triglycerides and lower levels of high-density lipoproteins are reported in these patients. *Conclusions*: We suggest performing liver US screening in subjects affected by IBD to detect NAFLD earlier. Also, patients with NAFLD present several metabolic comorbidities that would fall within the new definition of metabolic-associated fatty liver disease. Finally, we encourage larger longitudinal studies, including healthy controls, to provide further confirmation of our preliminary data.

## 1. Introduction

### 1.1. Crosstalk between IBD and NAFLD

Inflammatory bowel disease (IBD) is an inflammatory condition encompassing two major forms: Crohn’s disease (CD) and ulcerative colitis (UC). They are characterized by an unregulated and abnormal immune response induced by environmental stimuli in genetically predisposed subjects [1]. In about 5–50% of patients with IBD, there are several extraintestinal manifestations such as musculoskeletal, ocular, cutaneous, and hepatobiliary. Hepatobiliary manifestations include primary sclerosing cholangitis, autoimmune/granulomatous hepatitis, and in particular, nonalcoholic fatty liver disease (NAFLD) [2,3]. NAFLD is currently the main cause of chronic liver disease in the general population worldwide and ranges from simple fatty liver to steatohepatitis to advanced fibrosis and finally cirrhosis [4,5]. It can be considered a manifestation of metabolic syndrome often associated with obesity, insulin resistance, dyslipidemia, and hypertension [6,7,8]. The prevalence of NAFLD in IBD patients is broadly variable due to different diagnostic methodologies and ranges from 20–30% of patients identified using hepatic ultrasound (US) to 24% of individuals diagnosed by magnetic resonance enterography to 71% of cases with transient elastography [9,10,11]. The overall prevalence is approximately 32% and, thus, considerably higher than the general population rate (25.2%) [12]. Despite a large number of studies, the pathogenetic mechanisms related to the onset of steatosis and the development of liver damage in patients with IBD are not entirely understood. Also, other risk factors can be involved in this association, such as chronic inflammation, drug-induced liver injury, prolonged steroid exposure, malnutrition, and gut dysbiosis [13,14]. In the genetic field, a previous study has shown how patients with IBD carrying the p.I148M missense variant in the patatin-like phospholipase domain-containing protein 3 (PNPLA3) gene, an important common genetic determinant of liver fat content and progression to chronic liver disease, have higher susceptibility to hepatic steatosis and liver damage [15]. A more recent cross-sectional study by Rodriguez-Duque et al. on 838 IBD patients compared with 1718 controls showed that these patients are at higher risk of developing fatty liver, not only for their weight or the presence of hypertension, diabetes, or high cholesterol but also for variables related to intestinal disease, such as IBD duration, activity, and prior surgery, that can be considered major predictors of incident NAFLD [16,17,18,19].

### 1.2. Diagnostic Approaches in NAFLD

Liver involvement associated with NAFLD in IBD patients complicates therapeutic management and increases the risk of hospitalization and mortality [20]. Thus, it is essential to adopt an appropriate diagnostic approach aimed at identifying and staging early NAFLD in IBD patients. Specifically, Hamaguchi’s score operates with an abdominal US scoring system to provide accurate indications of hepatic steatosis, visceral obesity, and metabolic syndrome [21]. The diagnosis of NAFLD requires hepatic fat assessment by imaging techniques or histology, excluding other causes of secondary fat accumulation (e.g., use of alcohol or steatogenic drugs) [22]. The gold standard in the diagnosis of NAFLD is liver biopsy, but it is an invasive and not very reproducible as well as expensive technique [23]. At the same time, the use of transient elastography makes it possible to determine liver stiffness and quantify steatosis using controlled attenuation parameters with high accuracy, but it is not accessible worldwide. Furthermore, it requires technical expertise and is unreliable in patients with severe obesity and ascites [24,25]. Therefore, US examination is the most common item performed in clinical practice for the diagnosis of NAFLD [26]. However, although US is an easily reproducible and inexpensive technique, it has high interindividual variability [27]. Currently, US data on NAFLD in IBD patients are quite heterogeneous. A recent meta-analysis showed a prevalence with different imaging techniques of 30% and that the risk of NAFLD was two times higher in IBD patients versus healthy subjects [28]. Another study showed the US prevalence of NAFLD in IBD patients treated with biological therapy at 54% [29]. Similar results were obtained by Shintaku et al., with a US prevalence of NAFLD of 45% among 71 enrolled IBD patients [30]. In addition, due to the newly proposed nomenclature of metabolic-associated fatty liver disease (MAFLD), there is a need for continuous evaluation of the clinical features of these patients, especially from a metabolic perspective [7].

### 1.3. Aims

This cross-sectional study aimed to evaluate the US prevalence of NAFLD in patients with IBD and to evaluate their clinical features.

## 2. Materials and Methods

### 2.1. Patients

We retrospectively enrolled 143 patients with clinical, endoscopic, and radiological diagnoses of IBD [1]. According to specific inclusion criteria: (i) patients of age ≥18, (ii) patients subjected to hepatic US at hospital admission; and exclusion criteria: (i) patients with a history of alcohol or drug abuse, (ii) patients with previous or current viral hepatitis infection, (iii) patients with autoimmune liver disease, (iv) cirrhotic patients, (v) patients with malignancies, (vi) pregnant and/or lactating women. From each patient were collected (i) demographic and anthropometric data, (ii) disease characteristics, (iii) disease location and phenotype, (iv) dysmetabolic comorbidities, (v) laboratory parameters, and (vi) medications.

### 2.2. NAFLD Diagnosis

All patients underwent liver evaluation by US, according to a previous study by Mancina et al. [15]. Briefly, abdominal US was performed by the same experienced operator with a grayscale scanner device (LOGIQ S8 XDclear 2.0+, GE HealthCare, Milan, Italy) using a 3.5-MHz convex transducer with B-mode image evaluation. Individuals were fasting at least 4 h before the procedure. Before the procedure, the subjects followed a fiber-free diet and took 80 mg of simethicone thrice daily for 3 days. Hepatic steatosis was graded as mild (steatosis grade 1 or S1), moderate (steatosis grade 2 or S2), or severe (steatosis grade 3 or S3). Mild liver steatosis (S1) features were defined as a slight increase in liver echogenicity with a slight exaggeration of liver and kidney echo discrepancy. Moderate liver steatosis (S2) features were defined as an increase in liver echogenicity and loss of echoes from the wall of the portal vein with a greater posterior beam attenuation and greater discrepancy between hepatic and renal echoes. The features of severe liver steatosis (S3) were defined as a greater reduction in beam penetration, loss of echoes from most of the portal vein wall, and an even larger discrepancy between hepatic and renal echoes. Hepatic steatosis was defined as a steatosis grade of ≥S1 [27,31,32].

Anamnestic, laboratory, and endoscopic data were also collected. If laboratory and endoscopic data were not available, data resulting from investigations carried out on another date, ranging from 15 days before or after the date of the hepatic US, were used.

### 2.3. Study Design

Patients were stratified according to the presence or absence of hepatic fat accumulation at US examination. We chose to adopt additional exclusion criteria (self-defined plus) to identify which patients had liver steatosis not attributable to dysmetabolic comorbidities: obesity, high waist circumference, insulin resistance, type 2 diabetes mellitus (T2DM), hypertension, and dyslipidemia. Application of these criteria allows the evaluation of hepatic steatosis independently from factors attributable to metabolic syndrome. This approach was similarly applied in a previous study by Angelico et al. [33].

### 2.4. Statistical Analysis

We reported quantitative variables as mean ± standard deviation (SD) and nominal variables as percentages and absolute numbers. Comparisons of continuous variables were performed by the Student’s *t*-test or the Mann–Whitney U test, considering each quantitative trait after testing it for normality using the Shapiro–Wilk test. Differences between categorical variables were assessed by the chi-square (χ^2^) test. A *p*-value < 0.05 was considered statistically significant. The data were analyzed using SPSS 26.0 software (IBM Corp., Armonk, NY, USA).

### 2.5. Ethics

This study was approved by the local ethics committee of Magna Graecia University (protocol number 2014/49). This study was conducted in compliance with the principles outlined in the Declaration of Helsinki. Informed written consent was obtained from each participating patient.

## 3. Results

### 3.1. Characteristics of the Patients Enrolled

**Figure 1:**
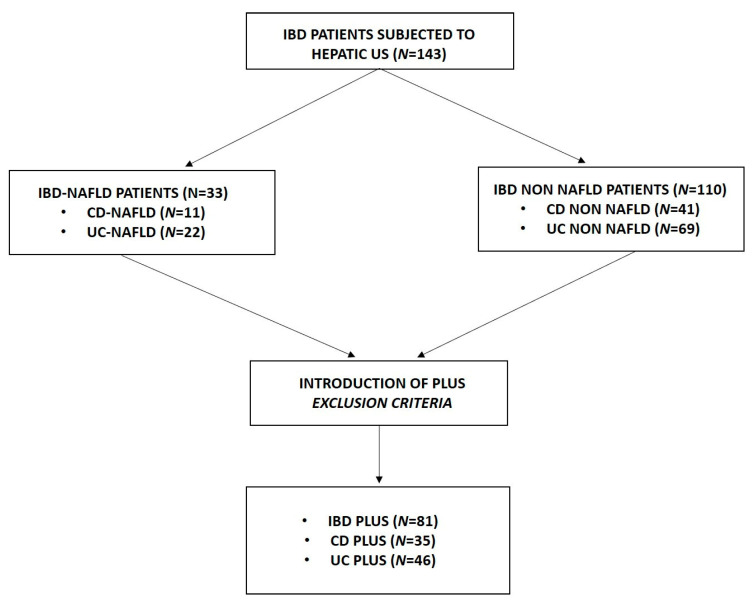
Workflow of study design. Patients were enrolled in the study and divided into different groups.

All 143 IBD patients were subjected to hepatic US to assess the presence of steatosis. Among them, 33 patients (11 with CD and 22 with UC, respectively) showed hepatic steatosis, while 110 patients (41 with CD and 69 with UC, respectively) did not show hepatic steatosis. Subsequently, self-defined plus exclusion criteria were applied, obtaining 81 IBD patients, divided into 35 patients with CD and 46 with UC (Figure 1).

### 3.2. Comparison between IBD Patients

The main clinical and laboratory features of the subjects enrolled in our study are summarized in Table 1. Most IBD patients under investigation were males (*n* = 82, 57%), with a mean age of 45 ± 16 years and a body mass index (BMI) and waist circumference of 25 ± 4 kg/m^2^ and 91 ± 12 cm, respectively. Most UC patients showed mild or severe liver steatosis (*n* = 18, 20% and *n* = 2, 2%, respectively). On the other hand, CD showed a higher percentage of moderate liver steatosis (*n* = 4, 8%). Ninety-one (63%) patients had UC, and most of them (*n* = 48, 54%) extended to the entire colon. Fifty-two (37%) had CD, and most of them with an ileal and ileocolonic extension: 41% and 42%, respectively. About 40% of CD patients (*n* = 21) had a stenosing disease phenotype. Twenty-four (17%) had previously undergone surgery. UC patients showed more dysmetabolic comorbidities than CD patients but similar levels on laboratory parameters. IBD patients were treated with salicylate (*n* = 75, 52%), azathioprine (*n* = 47, 33%), and biological therapy (*n* = 86, 60%).

### 3.3. US Prevalence of NAFLD among IBD Patients

**Figure 2:**
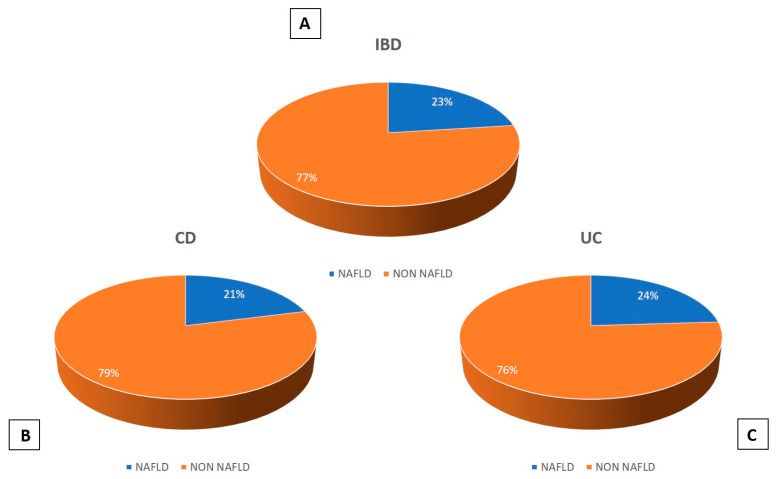
(**A**) US prevalence of NAFLD in IBD patients; (**B**) US prevalence of NAFLD in patients with CD; (**C**) US prevalence of NAFLD in patients with UC.

The US prevalence of NAFLD was 23%, considering the unified sample (IBD): 21% and 24% in CD and UC, respectively (Figure 2).

### 3.4. Comparison between IBD Patients with and without NAFLD

As shown in Table 2, subjects with IBD were stratified according to the ultrasonographic of NAFLD.

Most IBD–NAFLD patients were males (*n* = 24 (73%) vs. *n* = 58 (53%), *p* = 0.047) and showed significantly higher values than IBD non-NAFLD patients for age (53 ± 13 vs. 43 ± 17 years, *p* = 0.03), BMI (27 ± 5 vs. 24 ± 4 kg/m^2^, *p* < 0.001), and waist circumference (100 ± 11 vs. 88 ± 11 cm, *p* < 0.001). None of the IBD–NAFLD patients was an active smoker (*n* = 0 vs. *n* = 4 (4%), *p* = 0.266). Furthermore, a significantly higher percentage of IBD–NAFLD patients reported hypertension (*n* = 13 (39%) vs. *n* = 11 (10%), *p* < 0.001) and IBD plus dysmetabolic criteria (*n* = 26 (78%) vs. *n* = 36 (33%), *p* < 0.001). No significant differences between the two groups for type 2 diabetes mellitus (T2DM; *n* = 4 (12%) vs. *n* = 7 (7%), *p* = 0.278) and dyslipidemia (*n* = 5 (15%) vs. *n* = 13 (12%), *p* = 0.564) were found. Regarding laboratory parameters, IBD–NAFLD patients showed significantly higher values of alanine aminotransferase (ALT; 22 ± 10 vs. 18 ± 9 UI/L, *p* = 0.034) and triglycerides (123 ± 63 vs. 93 ± 40 mg/dL, *p* = 0.002) but significantly lower values of high-density lipoproteins (HDL; 48 ± 16 vs. 58 ± 17 mg/dL, *p* = 0.005). No significant differences were found between the two groups for the other laboratory parameters. IBD–NAFLD patients showed a higher disease duration (15 ± 10 vs. 11 ± 9 years, *p* = 0.044) and age at onset (38 ± 16 vs. 32 ± 15 years, *p* = 0.047) than IBD non-NAFLD patients. Most IBD–NAFLD patients had UC (*n* = 22 (66%) vs. *n* = 69 (63%), *p* = 0.830), while in the other group, there was a higher percentage of CD patients (*n* = 41 (37%) vs. *n* = 11 (33%), *p* = 0.837). Among IBD patients, there was a significant difference between groups for the stenosing phenotype (*n* = 7 (64%) vs. *n* = 12 (29%), *p* = 0.035) and left-side colitis (*n* = 7 (32%) vs. *n* = 9 (13%), *p* = 0.044). There was no significant difference between groups for the other disease locations and phenotypes. Otherwise, IBD non-NAFLD patients showed a higher Harvey–Bradshaw Index (7 ± 3 vs. 5 ± 2, *p* = 0.033) than IBD patients with NAFLD. In addition, none of IBD patients treated with vedolizumab showed NAFLD (*n* = 0 vs. *n* = 16 (23%), *p* = 0.023), while most IBD–NAFLD patients were treated with antitumor necrosis factor-alfa (TNF-α; *n* = 15 (88%) vs. *n* = 46 (67%), *p* = 0.841) and ustekinumab (*n* = 2 (12%) vs. *n* = 7 (10%), *p* = 1.000) but with no statistically significant difference between the two groups. Finally, regarding the other medications, most IBD–NAFLD patients were treated with salicylate (*n* = 20 (61%) vs. *n* = 55 (50%), *p* = 0.324), azathioprine (*n* = 10 (30%) vs. *n* = 37 (33%), *p* = 0.834), or were undergoing surgery (*n* = 7 (21%) vs. *n* = 17 (15%), *p* = 0.435), with no significant differences between groups.

## 4. Discussion

NAFLD is frequently associated with IBD: both metabolic features and intestinal inflammation are involved in the pathogenesis of IBD-associated NAFLD. In this context, our study showed a US prevalence of NAFLD of 23% in IBD patients. These data are in line with recent epidemiological investigations that showed a US prevalence of 20–50% [12,29,30,34]. Given that IBD is a risk factor for NAFLD, this result underlines the importance of performing hepatic US in at-risk patients. Moreover, this approach should be applied in clinical practice not only to patients with IBD but also to other risk categories. In addition, due to its low cost, it should be used to follow disease progress over time [8]. In this complex interplay between genetic, metabolic, inflammatory, and pharmacological factors, the existing causative relationship and the underlying pathogenic mechanisms that might recognize the gut microbiota as a key link remain unclear [10]. Most IBD–NAFLD patients were male with an older age and age at onset than IBD patients without NAFLD. These data are explained by longer disease duration in patients with liver steatosis and were confirmed by Sourianarayanane et al., who described NAFLD patients as older (46.0 ± 13.3 vs. 42.0 ± 14.1 years; *p* = 0.018) and with a later onset of IBD compared with the control group (37.2 ± 15.3 vs. 28.7 ± 23.8 years; *p* = 0.002) [35]. These data were confirmed by Glassner et al., who showed that IBD–NAFLD patients had significantly longer disease duration than IBD-only patients (20 ± 12.2 vs. 10 ± 7.7 years, *p* = 0.004) [6]. Longer disease duration may lead to several risk factors for NAFLD, such as chronic inflammation and dysbiosis of the gut microbiota. It is plausible that gut dysbiosis may play a pivotal role in the biochemical and metabolic pathways that correlate with the onset and progression of IBD-associated NAFLD [5]. No significant difference was found for the number of relapses, extraintestinal manifestations, and active disease, according to Scrivo et al. [36]. At the same time, the IBD–NAFLD patients of our cohort showed a significantly higher BMI and waist circumference and a significantly higher percentage of hypertension than IBD non-NAFLD patients. These findings, along with the significantly lower HDL and higher triglycerides levels in patients with liver steatosis, support the use of new MAFLD nomenclature, which includes in the definition of NAFLD the additional dysmetabolic comorbidities we investigated in this study and additional risk factors, such as genetics and environmental factors and gut dysbiosis [7,37]. Our data are in agreement with the study by Magri et al. on a cohort of patients with characteristics similar to the present investigation. Indeed, NAFLD patients showed higher BMI and waist circumference vs. non-NAFLD patients. Furthermore, additional parameters such as visceral and body fat were evaluated. In this regard, the percentage of visceral fat was higher in NAFLD patients [38]. Hoffmann et al. also confirmed this evidence in their monocentric retrospective study performed on 153 IBD patients [39]. In addition, confirming this evidence, Saroli Palumbo et al. indicated how extrahepatic diseases such as chronic kidney disease and cardiovascular diseases are more common among IBD–NAFLD patients [40]. As expected, ALT levels were significantly higher in IBD–NAFLD patients than in the IBD non-NAFLD group. Indeed, liver enzyme levels and BMI are robust predictors of the risk of NAFLD in IBD [9]. Among IBD patients, the percentage of left-side colitis in UC was significantly higher in patients with liver steatosis vs. the non-NAFLD group. This finding is consistent with a previous study showing that a more extensive disease and a higher number of annual relapses and surgeries correlate with more severe steatosis [41]. On the other hand, the stenosing phenotype percentage in CD patients was significantly higher in NAFLD patients than in non-NAFLD. The possible reason is that our cohort is characterized by patients with long-term disease, who are thus more likely to have a more severe phenotype and, consequently, a greater susceptibility to liver steatosis [42]. Furthermore, recent studies have investigated whether CD is a stronger risk factor for developing NAFLD than UC. However, this hypothesis remains to be investigated because of the many genetic, environmental, and metabolic factors that play a major role in the establishment of hepatic steatosis in IBD patients [43]. Regarding biological therapy, vedolizumab was the only biological drug with a significant statistical difference between IBD–NAFLD patients and IBD non-NAFLD patients. In the literature, an antihepatic steatosis effect of anti-TNF-α treatments has been suggested [6], while there is no evidence of this effect for vedolizumab [44,45]. However, given the cross-sectional nature of this study, investigation of the mechanistic role of biological therapy in IBD–NAFLD patients falls beyond the purpose of our aims. Finally, 78% of the IBD population showed NAFLD without additional metabolic features (IBD plus dysmetabolic criteria). This high number is now first described, considering that the “multiple-hit hypothesis” of NAFLD includes as risk factors several comorbidities analyzed in this study, namely, obesity, insulin resistance, T2DM, and dyslipidemia [46]. Our data provide an analysis of the prevalence and clinical features of NAFLD in IBD patients admitted to a real-life hospital setting. This is one of the few observational studies in the literature that describes the clinical features of liver steatosis in IBD patients with and without dysmetabolic comorbidities.

The main limitations of our study are the small number of patients involved and the absence of a healthy control group. The latter is an interesting point as 25–30% of healthy people can have liver steatosis findings at screening abdominal US [6]. Thus, controlled trials in this field are needed to confirm these results.

## 5. Conclusions

Our data suggest the importance of performing US examinations in patients with IBD to detect NAFLD as early as possible. This clinical strategy can be central in improving the management of subjects affected by both these conditions. In addition, patients with NAFLD present several metabolic comorbidities that would fall within the new definition of MAFLD. Our preliminary results invite further confirmation on larger longitudinal studies including healthy controls.

## Figures and Tables

**Table 1:** Clinical and laboratory features of patients.

	IBD (*N* = 143)	CD (*N* = 52)	UC (*N* = 91)
Demographic and Anthropometric			
Age (years)	45 ± 16	44 ± 17	45 ± 15
Male gender, *n* (%)	82 (57)	31 (60)	51 (56)
Active smoker, *n* (%)	4 (3)	3 (6)	1 (1)
BMI (kg/m^2^)	25 ± 4	24 ± 4	25 ± 5
Waist circumference (cm)	91 ± 12	89 ± 11	91 ± 13
Disease characteristic			
Disease duration (years)	12 ± 9	13 ± 9	11 ± 10
Age at onset (years)	33 ± 15	34 ± 13	32 ± 14
CD (Harvey–Bradshaw index)	-	7 ± 3	-
UC (full Mayo Score)	-	-	2 ± 0.7
Relapse/year	1.3 ± 0.7	1.3 ± 0.9	1.2 ± 0.7
Active disease, *n* (%)	47 (33)	27 (52)	20 (22)
Extraintestinal manifestations, *n* (%)	26 (18)	13 (25)	13 (14)
NAFLD, *n* (%)	33 (23)	11 (21)	22 (24)
Mild steatosis, *n* (%)	24 (17)	6 (11)	18 (20)
Moderate steatosis, *n* (%)	6 (4)	4 (8)	2 (2)
Severe steatosis, *n* (%)	3 (2)	1 (2)	2 (2)
Surgery, *n* (%)	24 (17)	17 (33)	7 (8)
CD disease location and phenotype, *n* (%)			
Ileal	-	21 (41)	-
Colonic	-	8 (15)	-
Ileo–colonic	-	22 (42)	-
Upper GI	-	1 (2)	-
Inflammatory	-	16 (31)	-
Fistulizing	-	15 (29)	-
Stenosing	-	21 (40)	-
UC disease location, *n* (%)			
Proctitis	-	-	8 (8)
Proctosigmoiditis	-	-	19 (21)
Left side	-	-	16 (17)
Pancolitis	-	-	48 (54)
Dysmetabolic comorbidities, *n* (%)			
T2DM	11 (8)	1 (2)	10 (11)
Hypertension	24 (17)	8 (15)	16 (18)
Dyslipidemia	18 (13)	5 (10)	13 (14)
IBD plus dysmetabolic criteria	81 (57)	35 (67)	46 (50)
Laboratory parameters			
ALT (UI/L)	19 ± 10	18 ± 9	20 ± 11
AST (UI/L)	20 ± 9	22 ± 9	21 ± 10
Total cholesterol (mg/dL)	168 ± 42	167 ± 41	168 ± 43
LDL (mg/dL)	104 ± 35	103 ± 34	105 ± 35
HDL (mg/dL)	56 ± 17	55 ± 17	56 ± 16
Triglycerides (mg/dL)	99 ± 48	99 ± 49	100 ± 48
Fasting blood glucose (mg/dL)	88 ± 20	87 ± 19	89 ± 21
Fasting insulinemia (mg/dL)	10 ± 7	10 ± 8	9 ± 8
HOMA-IR	2 ± 2	2 ± 1	2 ± 3
Fecal calprotectin (mcg/gr)	501 ± 797	492 ± 802	509 ± 804
Medication, *n* (%)			
Salicylates, *n* (%)	75 (52)	24 (46)	51 (56)
Azathioprine, *n* (%)	47 (33)	16 (31)	31 (34)
>3 cycles of steroids, *n* (%)	34 (24)	9 (17)	25 (27)
Biological therapy, *n* (%)	86 (60)	33 (63)	53 (58)
Anti-TNF-α, *n* (%)	61 (71)	25 (76)	36 (68)
Vedolizumab, *n* (%)	16 (19)	2 (6)	14 (26)
Ustekinumab, *n* (%)	9 (10)	6 (18)	3 (6)
>1 Biological drug, *n* (%)	23 (16)	4 (8)	19 (21)
Current biological therapy duration (years)	3 ± 2	4 ± 2	2 ± 2
Total biological therapy duration (years)	5 ± 4	4 ± 3	4 ± 2

Legend: IBD, inflammatory bowel disease; CD, Crohn’s disease; UC, ulcerative Colitis; BMI, body mass index; NAFLD, nonalcoholic fatty liver disease; T2DM, type 2 diabetes mellitus; ALT, alanine aminotransferase; AST, aspartate aminotransferase; LDL, low-density lipoprotein; HDL, high-density lipoprotein; HOMA-IR, homeostasis model assessment of insulin resistance; TNF-α, tumor necrosis factor-alfa.

**Table 2:** Comparison between IBD patients stratified for NAFLD and non-NAFLD.

	IBD–NAFLD (*N* = 33)	IBD Non-NAFLD (*N* = 110)	*p*-Value
Demographic and Anthropometric			
Age (years)	53 ± 13	43 ± 17	0.03
Male gender, *n* (%)	24 (73)	58 (53)	0.047
Active smoker, *n* (%)	0	4 (4)	0.266
BMI (kg/m^2^)	27 ± 5	24 ± 4	<0.001
Waist circumference (cm)	100 ± 11	88 ± 11	<0.001
Disease characteristic			
Disease duration (years)	15 ± 10	11 ± 9	0.044
Age at onset (years)	38 ± 16	32 ± 15	0.047
CD, *n* (%)	11 (33)	41 (37)	0.837
UC, *n* (%)	22 (66)	69 (63)	0.830
CD (Harvey–Bradshaw index)	5 ± 2	7 ± 3	0.033
UC (full Mayo Score)	2 ± 0.6	2 ± 0.8	0.612
Relapse/year	1.3 ± 0.7	1.3 ± 0.8	1.000
Active disease, *n* (%)	12 (36)	35 (32)	0.675
Extraintestinal manifestations, *n* (%)	7 (21)	19 (17)	0.612
Surgery, *n* (%)	7 (21)	17 (15)	0.435
CD disease location and phenotype, *n* (%)			
Ileal *	3 (27)	18 (44)	0.30
Colonic *	1 (9)	7 (17)	0.51
Ileo–colonic *	7 (64)	15 (37)	0.106
Upper GI *	0	1 (2)	0.60
Inflammatory *	1 (9)	16 (39)	0.06
Fistulizing *	3 (27)	13 (32)	0.70
Stenosing *	7 (64)	12 (29)	0.035
UC disease location, *n* (%)			
Proctitis *	0	8 (12)	0.09
Proctosigmoiditis *	2 (9)	17 (25)	0.11
Left side *	7 (32)	9 (13)	0.044
Pancolitis *	13 (59)	35 (50)	0.42
Dysmetabolic comorbidities, *n* (%)			
T2DM	4 (12)	7 (7)	0.278
Hypertension	13 (39)	11 (10)	<0.001
Dyslipidemia	5 (15)	13 (12)	0.564
IBD plus dysmetabolic criteria	26 (78)	36 (33)	<0.001
Laboratory parameter			
ALT (UI/L)	22 ± 10	18 ± 9	0.034
AST (UI/L)	22 ± 10	20 ± 9	0.187
Total cholesterol (mg/dL)	168 ± 47	167 ± 40	0.994
LDL (mg/dL)	107 ± 38	103 ± 33	0.591
HDL (mg/dL)	48 ± 16	58 ± 17	0.005
Triglycerides (mg/dL)	123 ± 63	93 ± 40	0.002
Fasting blood glucose (mg/dL)	92 ± 25	87 ± 18	0.156
Fasting insulinemia (mg/dL)	10 ± 8	10 ± 7	0.106
HOMA-IR	3 ± 2	2 ± 2	0.078
Fecal calprotectin (mcg/gr)	439 ± 911	519 ± 764	0.613
Medication, *n* (%)			
Salicylates, *n* (%)	20 (61)	55 (50)	0.324
Azathioprine, *n* (%)	10 (30)	37 (33)	0.834
>3 cycles of steroids, *n* (%)	9 (27)	25 (23)	0.643
Biological therapy, *n* (%)	17 (51)	69 (63)	0.311
Anti-TNF-α, *n* (%)	15 (88)	46 (67)	0.841
Vedolizumab, *n* (%)	0	16 (23)	0.023
Ustekinumab, *n* (%)	2 (12)	7 (10)	1.000
>1 Biological drug, *n* (%)	5 (15)	18 (16)	1.000
Current biological therapy duration (years)	4 ± 3	3 ± 2	0.188
Total biological therapy duration (years)	5 ± 3	4 ± 3	0.251

Legend: IBD, inflammatory bowel disease; CD, Crohn’s disease; UC, ulcerative colitis; BMI, body mass index; NAFLD, nonalcoholic fatty liver disease; T2DM, type 2 diabetes mellitus; ALT, alanine aminotransferase; AST, aspartate aminotransferase; LDL, low-density lipoprotein; HDL, high-density lipoprotein; HOMA-IR, homeostasis model assessment of insulin resistance; TNF-α, tumor necrosis factor-alfa. * The *p*-value was evaluated with regard to CD and UC patients, respectively.

## Data Availability

The data presented in this study are available on request from the corresponding author.
